# Characterisation of the First Enzymes Committed to Lysine Biosynthesis in *Arabidopsis thaliana*

**DOI:** 10.1371/journal.pone.0040318

**Published:** 2012-07-05

**Authors:** Michael D. W. Griffin, Jagan M. Billakanti, Akshita Wason, Sabrina Keller, Haydyn D. T. Mertens, Sarah C. Atkinson, Renwick C. J. Dobson, Matthew A. Perugini, Juliet A. Gerrard, Frederick Grant Pearce

**Affiliations:** 1 Department of Biochemistry and Molecular Biology, Bio21 Molecular Science and Biotechnology Institute, University of Melbourne, Melbourne, Victoria, Australia; 2 Biomolecular Interactions Centre and School of Biological Sciences, University of Canterbury, Christchurch, New Zealand; 3 Industrial Research Limited, Lower Hutt, New Zealand; 4 Australian Synchrotron, Melbourne, Victoria, Australia; 5 Department of Biochemistry, La Trobe Institute for Molecular Science, La Trobe University, Melbourne, Victoria, Australia; Institute of Enzymology of the Hungarian Academy of Science, Hungary

## Abstract

In plants, the lysine biosynthetic pathway is an attractive target for both the development of herbicides and increasing the nutritional value of crops given that lysine is a limiting amino acid in cereals. Dihydrodipicolinate synthase (DHDPS) and dihydrodipicolinate reductase (DHDPR) catalyse the first two committed steps of lysine biosynthesis. Here, we carry out for the first time a comprehensive characterisation of the structure and activity of both DHDPS and DHDPR from *Arabidopsis thaliana*. The *A. thaliana* DHDPS enzyme (*At*-DHDPS2) has similar activity to the bacterial form of the enzyme, but is more strongly allosterically inhibited by (*S*)-lysine. Structural studies of *At*-DHDPS2 show (*S*)-lysine bound at a cleft between two monomers, highlighting the allosteric site; however, unlike previous studies, binding is not accompanied by conformational changes, suggesting that binding may cause changes in protein dynamics rather than large conformation changes. DHDPR from *A. thaliana* (*At*-DHDPR2) has similar specificity for both NADH and NADPH during catalysis, and has tighter binding of substrate than has previously been reported. While all known bacterial DHDPR enzymes have a tetrameric structure, analytical ultracentrifugation, and scattering data unequivocally show that *At*-DHDPR2 exists as a dimer in solution. The exact arrangement of the dimeric protein is as yet unknown, but *ab initio* modelling of x-ray scattering data is consistent with an elongated structure in solution, which does not correspond to any of the possible dimeric pairings observed in the X-ray crystal structure of DHDPR from other organisms. This increased knowledge of the structure and function of plant lysine biosynthetic enzymes will aid future work aimed at improving primary production.

## Introduction

Lysine biosynthesis in plants provides an attractive target for the development of novel herbicides by inhibiting the pathway, or increasing the nutritional value of crops by increasing production of lysine [1]–[2]. Cereal crops have nutritionally limiting amounts of lysine, and work is ongoing to modify this metabolic pathway to increase the levels of this essential amino acid thus providing crops with higher nutritional value. Indeed, transgenic maize plants with increased lysine content have recently become commercially available [3]–[4].

Synthesis of lysine in plants uses the diaminopimelate (DAP) pathway, beginning with aspartate (Figure S1). While three established variants of the DAP pathway occur in prokaryotes, plants and photosynthetic cohorts use a novel variant of the pathway, in which (*S*)-tetrahydrodipicolinate (THDP) is converted directly into *LL*-DAP [5]–[6]. The first reaction specific to the DAP pathway is the condensation of (*S*)-ASA and pyruvate into HTPA by dihydrodipicolinate synthase (DHDPS), followed by the formation of THDP by dihydrodipicolinate reductase (DHDPR) (Figure S1). These two enzymes are common to all variants of the DAP pathway. In plants, DHDPS is located as a soluble stromal protein in the chloroplast [7], and studies of the DHDPS promoter show that it directs high expression in the meristems and vasculature of roots, stem and leaves [8].

**Figure 1:**
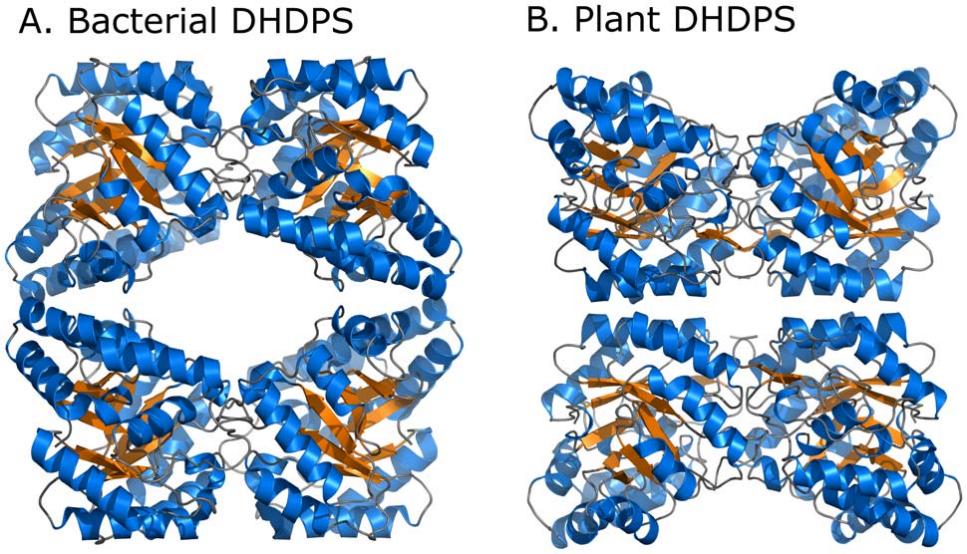
Comparison of bacterial and plant DHDPS. Structures shown for *E. coli* (1yxc) and *N. sylvestris* [16].

DHDPS has been extensively studied in bacteria and plants, including tobacco [7] (*Ns*-DHDPS), wheat [9]–[10], spinach [11], maize [12], pea [13], carrot [14] and grapevine [15]. In plants, DHDPS is inhibited by lysine, and thus is a key enzyme in regulating lysine biosynthesis. The structure of the tobacco enzyme shows that plant DHDPS is a homotetramer, made up of a dimer of tight dimers [16]. The active site is located at the centre of a (β/α)_8_-barrel in each monomer, with the lysine binding site located in a cleft at the tight-dimer interface. Most prokaryotic DHDPS enzymes have a similar homotetrameric structure, with the exception of *Staphylococcus aureus* (*Sa*-DHDPS) and *Pseudomonas aeruginosa* (*Pa*-DHDPS), which exist as dimers [17]–[18]. Curiously, while DHDPS from both plants and bacteria comprise a ‘dimer of dimers’ structure, they adopt a different configuration of dimers (Figure 1), leading to the hypothesis that each protein evolved from an ancestral dimeric enzyme [16], [19].

In comparison to DHDPS, DHDPR has been surprisingly unstudied in plants, with DHDPR from maize being the only plant DHDPR enzyme that has been well characterised [20]. The genes encoding plant DHDPR were only recently characterised, with the identification of two DHDPR orthologues in *Arabidopsis thaliana* [5]. Several bacterial DHDPR enzymes have been characterised, including those from *Escherichia coli* (*Ec*-DHDPR, pdb: 1arz) [21]–[22], *Mycobacterium tuberculosis* (*Mt*-DHDPR, pdb: 1c3v) [23], *Thermotoga maritima* (*Tm*-DHDPR, pdb: 1vm6) [24] and *S. aureus* (*Sa*-DHDPR, pdb: 3qy9) [25]–[27]. DHDPR catalyses the conversion of HTPA into THDP *via* a pyridine nucleotide linked reduction, and has dehydratase activity that initially converts HTPA into DHDP [28]. The *E. coli* and *M. tuberculosis* DHDPR enzymes are unusual in that they have similar specificity for both 2′-phosphorylated (NADPH) and non-phosphorylated (NADH) nucleotide substrates [21]–[23], while *T. maritima* and *S. aureus* DHDPR have a significantly higher affinity for NADPH [24]–[27]. All known bacterial DHDPR enzymes exist as homotetramers, with an N-terminal domain that binds to dinucleotides, and a C-terminal domain that binds to HTPA [23], [27], [29].

Genes encoding orthologues of DHDPS and DHDPR are located on chromosomes 2 (*At*-DHDPS1, *At*-DHDPR1) and 3 (*At*-DHDPS2, *At*-DHDPR2) of *A. thaliana*. The two isoforms of DHDPS show 84% identity at the nucleotide level, have similar functionality, and are both inhibited by (*S*)-lysine to a similar extent [30]. Complementation studies confirmed that the cDNAs encoding AT2G44040 and AT3G59890 were able to complement a DapB^−^ strain of *E. coli*, confirming that they encode for enzymes with DHDPR activity [5]. An additional gene with high homology to DHDPR (AT5G52100) did not complement the DapB^−^ strain, and was later shown to encode for chloroplast NAD(P)H dehydrogenase [31].

As part of our studies investigating enzymes involved in lysine biosynthesis [19], [24], [32]–[34], we have characterised the DHDPS and DHDPR enzymes from *A. thaliana*, in an effort to better understand the structure and function of two key enzymes involved in lysine biosynthesis in plants. The *At*-DHDPS2 isoform was chosen in order to complement previous studies which showed that the gene is expressed in the root apex and that mutations in the gene reduce lysine production [30], [35]. A detailed knowledge of these enzymes will assist in the design of novel herbicides aimed at inhibiting the lysine biosynthetic pathway, and will aid the development of crops that have higher nutritional value.

## Results and Discussion

Given the interest in (*S*)-lysine biosynthesis in plants as a target for improving nutritional value or as a target for pesticide development, we undertook a study of the first two enzymes of the pathway. No previous studies have characterised these enzymes together, or utilised improved methods for substrate preparation. Surprisingly, although more than 20 bacterial DHDPS structures have been determined to date, there have only been two plant DHDPS structures characterised [15]–[16], and little is known about the structure of plant DHDPR.

### Activity of *A. thaliana* DHDPS2

In order to better characterise the activity of one of the key regulatory steps of lysine biosynthesis, DHDPS2 from *A. thaliana* was cloned, expressed in *E. coli*, and purified to homogeneity. The enzyme is shown to be active, with a *K*_M_ for (*S*)-ASA of 0.09±0.01 mM, *K*_M_ for pyruvate of 1.0±0.1 mM, and *k*_cat_ of 93±5 s^−1^ (Figure S2). The reaction follows a ping-pong mechanism, in which pyruvate binds before (*S*)-ASA. Previous studies of plant DHDPS enzymes showed a *K*_M_(ASA) of 0.4–1.4 mM, and *K*_M_(pyruvate) of 1.7–12 mM [9]–[14]. The *K*_M_ values for *At*-DHDPS2 are lower than those previously reported for plant DHDPS, however all of the previous studies used (*S*)-ASA that was synthesised by ozonolysis, a method that has been shown to form inhibitory compounds that reduce the accuracy of the assay [36]. The previous studies also used a colorimetric assay that is less accurate over all conditions than the coupled assay used in this study. *At-*DHDPS2 is strongly inhibited by (*S*)-lysine, with a *K_0.5_* of 23±3 µM, which is similar to previously reported values [9]–[14], and consistent with its key role in regulating (*S*)-lysine biosynthesis.

### Structure of *A. thaliana* DHDPS2

**Figure 2:**
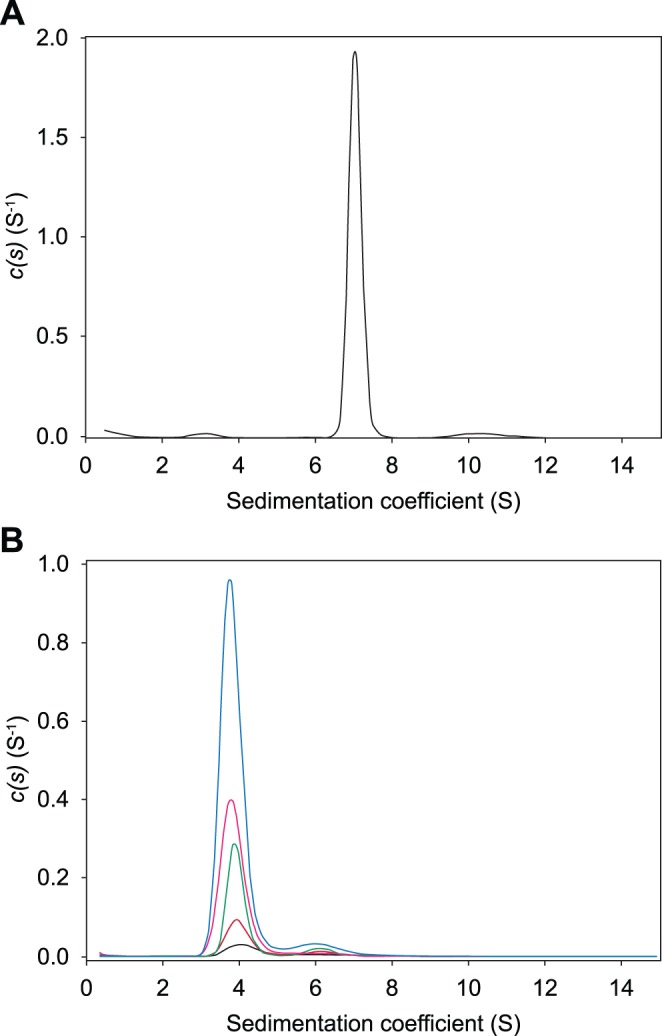
Analytical ultracentrifugation of *At*-DHDPS2 and *At*-DHDPR2. Sedimentation velocity analysis of *At*-DHDPS2 and *At*-DHDPR2. A) Continuous sedimentation coefficient distribution [*(c)s*] analysis of *At-*DHDPS2 at a concentration of 0.75 mg.mL^−1^ (black line). The RMSD and Runs Test Z (RTZ) scores for the fit were 0.008 and 3.2 respectively. B) *(c)s* analysis of *At-*DHDPR2 at concentrations of 0.1 mg.mL^−1^ (black line; RMSD = 0.009, RTZ = 2.4), 0.2 mg.mL^−1^ (red line; RMSD = 0.010, RTZ = 2.0), 0.4 mg.mL^−1^ (green line; RMSD = 0.014, RTZ = 8.6) 0.8 mg.mL^−1^ (pink line; RMSD = 0.013, RTZ = 4.9) and 1.6 mg.mL^−1^ (blue line; RMSD = 0.015, RTZ = 7.4). Radial absorbance data for the three lower protein concentrations were acquired at a different wavelength to those of the two highest protein concentrations, and the *c(s)* distributions were scaled accordingly. Residuals for the fits are shown in Figure S7.

**Figure 3:**
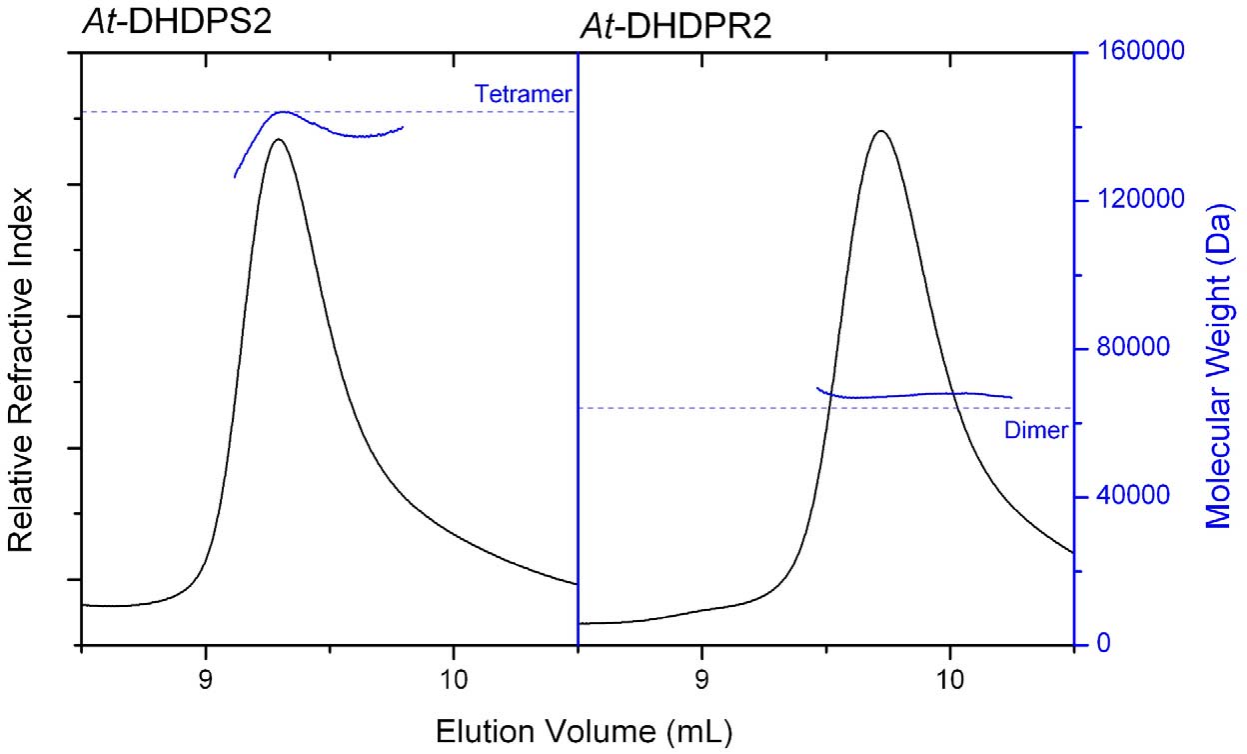
Light scattering analysis of *At*-DHDPS2 and *At*-DHDPR2 *At*-DHDPS2 and *At*-DHDPR2 were loaded onto a P3000 column and eluted using 20 mM Tris.HCl, 150 mM NaCl. Right angle light scattering and refractive index were measured and used to calculate the molecular weight, as described in the materials and methods. The dotted lines show the expected molecular weight calculated from the sequence for the dimeric and tetrameric enzymes.

Analytical ultracentrifugation and light scattering studies show that *At*-DHDPS2 exists as a homogenous tetramer in solution, with a modal sedimentation coefficient of 7.0 S and a molecular mass of approximately 140 kDa (Figures 2, 3), close to that expected for the tetramer. This compares well with previous studies that showed plant DHDPS enzymes adopt a homotetrameric structure [9], [11]–[12]. One study reported that the pea DHDPS enzyme may exist as a trimer [13], however this study was based on gel filtration studies which showed that pea DHDPS eluted with a similar molecular weight to maize, wheat and spinach, which have all been shown to be tetramers.

**Figure 4:**
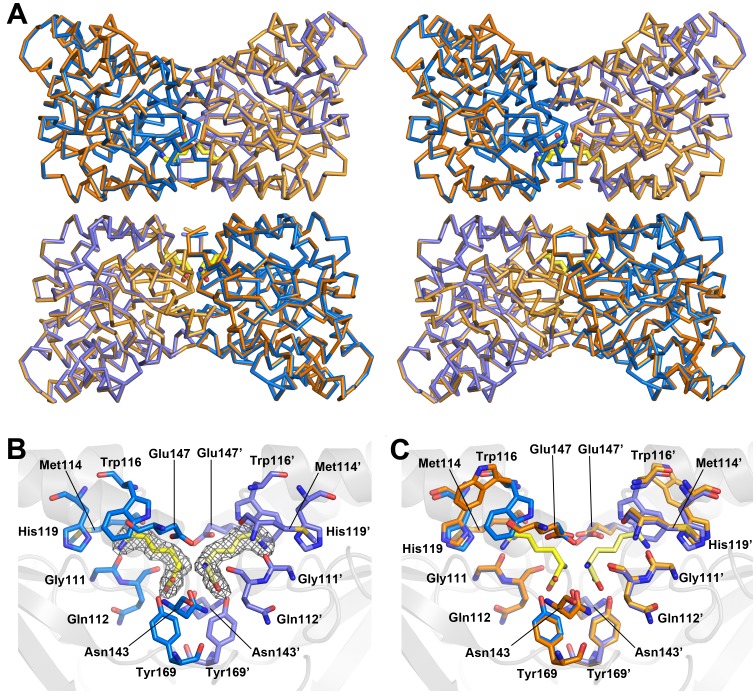
Crystal structures of unliganded and lysine bound *At*-DHDPS2. A) Wall-eyed stereo image of the Cα superposition of *At-*DHDPS2 with bound lysine (blue Cα trace) and unliganded *At-*DHDPS2 (gold Cα trace; rmsd = 0.3 Å). The lysine molecules bound at the allosteric site of each monomer of the tetramer are shown in yellow (stick representation). B) The lysine binding site at the monomer-monomer interface of the tight-dimer showing residues in contact with the bound lysine molecules (yellow). Electron density around the bound lysine (grey mesh, contoured at 1.0 sigma) was calculated using refined coordinates omitting the bound lysine molecules. Residues contributed by each monomer of the tight-dimer are shown in different shades of blue, and are indicated by the use of the prime (’) symbol. C) overlay of the lysine binding residues of the tight-dimer from the lysine bound (blue) and unliganded (gold) structures. Lysine molecules are shown in yellow. Residues contributed by each monomer of the tight-dimer are shown in different shades of blue or gold, and are indicated by the use of the prime (’) symbol.

In order to further study the structure of plant DHDPS, crystals were obtained for a truncated form of the *At-*DHDPS2 protein [37] in both the unliganded form, and with (S)-lysine bound at the allosteric regulation site. The crystals diffracted to 2.0 and 2.2 Å respectively, which is much greater resolution than the structure previously reported for *Ns-*DHDPS [16]. The general arrangement of the *At-*DHDPS2 subunits is identical to that previously reported for *N. sylvestris* DHDPS [16] and *V. vinifera* DHDPS [15], in which the enzyme exists as a dimer of dimers. In this configuration the active site of each monomer faces outward from the tetramer, while access to the allosteric, lysine binding site is *via* a small solvent filled channel formed at the dimer-dimer interface (Figure 4). Analysis of the structure using the EBI PISA server [38] showed an extensive dimer interface (1800 Å^2^, 15.5% of the total monomer surface), and a smaller interface between the dimers (609 Å^2^, 4.6% of the total monomer surface).

Plant DHDPS enzymes have a similar monomeric structure to the tetrameric bacterial DHDPS enzymes (Figure 1). However the crystal structure of *Ns*-DHDPS showed that this enzyme adopted a different quaternary arrangement, with an alternative interface between the dimers (Figure 1) [16]. This alternative quaternary architecture has also been observed recently for DHDPS from the grapevine plant, *V. vinifera* [15]. Consistent with these previous studies, our results show that this configuration may be a general feature of plant DHDPS enzymes. These alternative tetrameric configurations have led to the hypothesis that the ancestral form of the enzyme existed as a dimer, and that tetramerisation in either the plant or bacterial form improved stability and catalytic ability of the enzyme [15]–[16], [19], [39].

(*S*)-Lysine is an allosteric inhibitor of plant DHDPS, providing feedback regulation of lysine biosynthesis. Previous studies of *Ns-*DHDPS reported substantial changes in the structure of the enzyme in the presence of (*S*)-lysine that were proposed to result in communication between the subunits of the tetramer, providing an explanation for the increased sensitivity of *Ns*-DHDPS to allosteric inhibition by lysine [16]. To investigate the effect of (*S*)-lysine binding to the structure of *At*-DHDPS2, we performed crystal soaks with lysine, and compared the structures of the lysine bound and unbound forms. Electron density showed that (*S*)-lysine is bound in the same allosteric pocket previously observed for *Ns*-DHDPS and *Ec*-DHDPS (Figure 4B and 4C). The carboxyl group of (*S*)-lysine is coordinated by Tyr130 and Asn143, while the α-amino group contacts the main chain oxygen of Gln112, and Glu147 and Asn143 of the opposing subunit. Similar to the *Ns*-DHDPS structure, the ε-amino group contacts Trp116 and His119, but also the main chain oxygen of Gly111, rather than Gly141 in the *Ns*-DHDPS structure.

An overlay of the (*S*)-lysine bound and unbound structures showed a surprisingly low difference between the structures, with Cα RMSDs of only 0.29 Å and 0.30 for the superposition of the tight-dimers (i.e. the crystallographic asymmetrical unit) and the authentic tetramers, respectively. The main structural movements on binding of lysine were a rearrangement of the Tyr116, His119 and Ile120 side chains to accommodate the (S)-lysine molecule. Previous studies of the *Ec*-DHDPS and *Ns*-DHDPS enzymes observed a movement of Tyr169 (*Ec*: Y106, *Ns*: Y130) towards the carboxyl group of (*S*)-lysine upon inhibitor binding, which causes a change in the conformation of the catalytically important Tyr170 residue (*Ec*: Y107, *Ns*: Y131). However, this is not seen for *At*-DHDPS2. The improved resolution and quality of the crystal structures presented here over those reported for *Ns*-DHDPS provide greater detail and accuracy of the structural rearrangements of the enzyme upon (*S*)-lysine binding. While the mechanism of allosteric inhibition by (*S*)-lysine is uncertain, Arg199 (*Ec*: R138, *Ns*: R160) has been implicated in this process. Arg199 is located in helix α5 and binds to the carboxyl group of (*S*)-ASA [16], [40]–[41]. In the case of *At*-DHDPS2, binding of (*S*)-lysine caused only slight changes in the orientation of Arg199.

**Figure 5:**
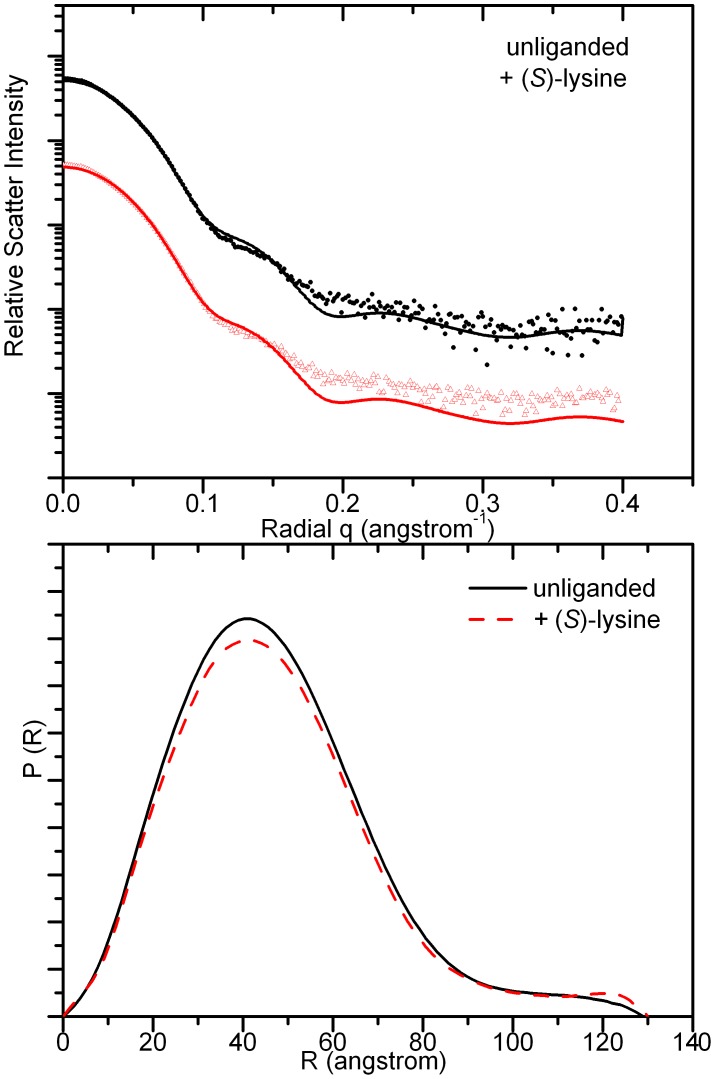
X-Ray scattering data of *At*-DHDPS2. Data were collected in the absence of ligand, or in the presence of 1 mM (S)-lysine, top panel; curves have been arbitrarily displaced along the logarithmic axis for clarity. Solid lines show the scattering profile from the unliganded crystal structure, calculated using CRYSOL. Distance-distribution functions, *p(r)* for the unbound and ligand bound *At*-DHDPS2 were determined using the indirect Fourier tranformation package GNOM (bottom panel).

To confirm that the crystal structures accurately reflect the structure of the protein in solution, small angle X-ray scattering data were collected for both free enzyme, and enzyme in the presence of (*S*)-lysine (Figure 5). The scattering data were compared to the theoretical scattering calculated from the crystal structure, and showed good agreement at low q (q <0.12 Å) corresponding to the overall shape for both the unliganded enzyme (χ = 0.91) and enzyme bound to (*S*)-lysine (χ = 1.20). Deviations from the fit at higher q, in particular, the smoothing out of the second maxima from the calculated profiles may result from flexibility of mobile regions, or perhaps small rearrangements of the quaternary structure in solution. The experimental scattering profile of *At*-DHDPS2 was unchanged by the addition of (*S*)-lysine or the substrate, pyruvate, showing that there are no large conformational changes in solution caused by ligand binding.

### Activity of *A. thaliana* DHDPR2

Since few studies have characterised DHDPR from plants, recombinant DHDPR2 from *A. thaliana* was cloned, expressed in *E. coli*, and purified to homogeneity. DHDPR catalyses the second reaction in the DAP pathway, namely the NAD(P)H-dependent reduction of HTPA (via dehydration first to DHDP) to form (S)-tetrahydrodipicolinate [28]. The enzyme was active in the presence of either NADPH or NADH, but showed inhibition by the HTPA substrate when NADH was used as a cofactor (Table 1, Figure S3). Maize DHDPR is the only plant DHDPR to have previously been characterised, and it was shown to have a *K*_M_(HTPA) of 430 µM, and *K*_M_(NADPH) of 46 µM [20]. These values are similar to the kinetic constants of 35 µM and 57 µM for NADPH and HTPA observed in this study, and those for bacterial DHDPR enzymes, which have a *K*_M_(HTPA) of 7.6 µM and *K*_M_(NADH) of 2.5 µM [24]. The difference in the Michaelis constant previously observed for HTPA may be a result of the inherent instability of HTPA in solution, or the presence of inhibitory compounds when (*S*)-ASA is produced by ozonolysis, as was used for the previous study, which has been shown to affect measurement of enzyme kinetics [36].

**Table 1:** Kinetic parameters (± SE) for *At*-DHDPR2 were calculated as described in materials and method section.

		NADH cofactor	NADPH cofactor
Constant NAD(P)H	*V*_max_ (µmol.s^−1^.mg^−1^)	388±95	881±66
	*K*_M_ (HTPA) (µM)	17±1	35±8
	*K*_iS_ (µM)	163±75	3300±900
Constant HTPA	*V*_max_ (µmol.s^−1^.mg^−1^)	310±20	1030±48
	*K*_M_ (NAD(P)H) (µM)	70±11	57±7

In order to determine whether DHDPS and DHDPR form a species-specific transient complex during catalysis, coupled assays were performed using different combinations of DHDPS and DHDPR enzymes from *T. maritima*, *E. coli* and *A. thaliana*. In all cases the measured activity of DHDPS was not dependent on the DHDPR used in the assay (data not shown), suggesting that there is no species specific coupling of DHDPS and DHDPR.

While *At*-DHDPR2 is similar to other DHDPR enzymes in having similar specificities for both NADH and NADPH [21], [23]–[24], [26]–[27], it shows a slightly higher catalytic rate in the presence of NADPH. Given that *At*-DHDPR2 is localised in the chloroplast [7], where NADPH is used as part of the light reactions, it is most likely that NADPH is the biologically relevant cofactor for *At*-DHDPR2. Substrate inhibition by HTPA has previously been observed for both *Tm*-DHDPR and *Sa-*DHDPR, and may be the result of a dead-end complex formed by HTPA binding to enzyme that has the oxidised form of nucleotide still bound to the active site [26]. During normal lysine synthesis in the plant, it is unlikely that HTPA would accumulate to sufficiently high levels for inhibition to occur, particularly when using NADPH as a cofactor.

### Structure of *A. thaliana* DHDPR

Structural studies of plant DHDPR have been extremely limited. All bacterial DHDPR enzymes characterised to date have been shown to exist as homotetramers, while maize DHDPR was reported to exist as a dimer [20]. In order to probe the quaternary structure of plant DHDPR, *At-*DHDPR2 was characterised using scattering studies and analytical ultracentrifugation.

*At*-DHDPS2 was eluted from a size-exclusion chromatography column, and measurement of right angle light scattering showed that the main peak of *At*-DHDPR2 was largely monodisperse, with a calculated molecular weight of 67.5 kDa. This is very similar to the expected molecular weight for a dimer of 64.0 kDa (Figure 3). A smaller peak (<5% of total peak area) was also observed, which was consistent with the size expected for a tetrameric species.

In order to better characterise *At*-DHDPR2 in solution, sedimentation velocity studies were carried out at 5 different protein concentrations (0.1–1.6 mg.mL^−1^) (Figure 2B). These experiments show two main species in solution, with the major peak having a sedimentation coefficient of ∼4 S, which corresponds to that expected for the dimer, and a minor peak with a sedimentation coefficient of ∼6.5 S, corresponding to that expected for a tetramer. The observation of a dimeric species has not previously been observed in studies of bacterial DHDPR enzymes [21]–[24], [26].

In bacteria, DHDPR is a homotetrameric enzyme, with each subunit consisting of two domains. The N-terminal domain includes a Rossman fold, and is the binding site for nucleotides, while the C-terminal domain includes the site for substrate and inhibitor binding [21]–[24], [29], [42]. *E. coli* DHDPR has an extensive interface between adjacent residues in which two 8-stranded β barrels are paired face to face to form a 16 stranded β barrel (Figure S4). One interface forms by pairing 4 strands of one subunit with 4 strands of an adjacent unit to form an 8-stranded mixed β sheet through pairing of β-10 (residues 229–238) of subunit A with subunit D [42]. An alpha helix, A4, is also involved in stabilising this subunit-subunit interaction through interactions between Val135, Val146, Met147 and Leu139. The other interface involves interactions between strand β-8 (residues 205–213), loop L1 (residues 195–203) and loop L2 (residues 164–167) of one subunit with the corresponding regions of the adjacent subunit. A similarly extensive interface has also been observed for *M. tuberculosis*, *T. maritima*, and *S. aureus* DHDPR enzymes [23]–[24], [27], as shown by analysing the interfaces of bacterial DHDPR structures using PISA (Table 2).

**Table 2:** Analysis of DHDPR interface regions using the PDBePISA web server (*38*).

	β-10 interface		β-8 interface	
Model	SISA(Å^2^)	# H-Bonds	SISA(Å^2^)	# H-Bonds
*E. coli* –1dih [42]	1563	26	1354	30
*E. coli –*1arz [29]	1519	21	1209	26
*T. maritima* –1vm6	1059	17	778	12
*S. aureus* –3qy9 [27]	1170	23	1339	23
*M. tuberculosis* –1c3v [23]	1127	24	1506	24
*M. tuberculosis* –1p9l [23]	1094	25	1487	23

If the plant DHDPR enzyme has a similar structural arrangement to bacterial DHDPR enzymes, the dimer observed by light scattering and analytical ultracentrifugation could correspond to that formed through the 8-stranded mixed β sheet (β-10 dimer), or that formed through interactions between strand β-8 and the extended loop region (β-8 dimer). Alignment of the protein sequences shows that *A. thaliana* (and other plants) have a truncation at the region corresponding to strand β-8, which may reduce the potential interactions at this interface (Figure S5).

**Figure 6:**
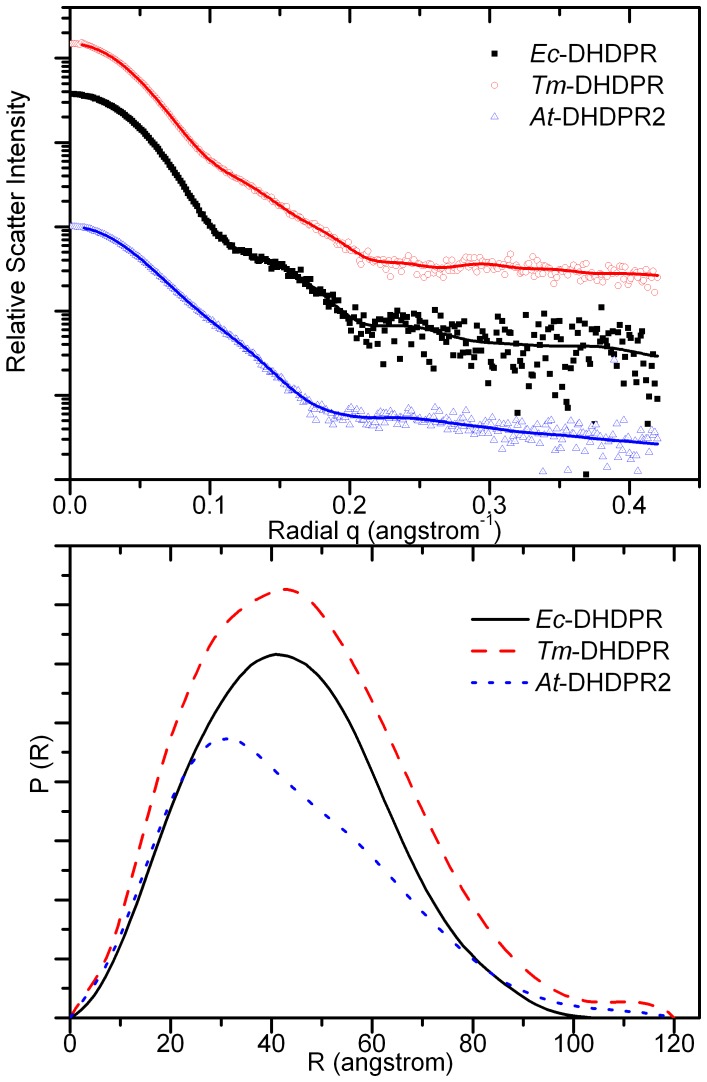
X-Ray scattering of DHDPR. Data were collected for *At*-DHDPR2, *Ec*-DHDPR and *Tm*-DHDPR (panel A); curves have been arbitrarily displaced along the logarithmic axis for clarity. Data was analysed using GNOM (fitted data shown by red line in panel A) to calculate a distance distribution function for each enzyme (panel B).

In order to gain information about the shape and structure in solution, small angle X-ray scattering data was collected for *At*-DHDPR2. *At*-DHDPR2 has a different scattering profile to that of *Ec*-DHDPR and *Tm*-DHDPR (Figure 6), and the real-space distance distributions, *p(r)* differ significantly. The near-symmetric *p(r)* of *Ec*-DHDPR and *Tm*-DHDPR are characteristic of spheroidal objects, whereas that of *At*-DHDPR2 is negatively skewed with a tail at long distances consistent with an elongated structure in solution. The particle volume and molecular weight estimated from the scattering data match the values expected for a dimeric enzyme in the case of *At*-DHDPR2, and a tetrameric enzyme in the case of *Ec*-DHDPR and *Tm*-DHDPR (Table 3). Comparison of the *At*-DHDPR2 scattering data with that calculated for the variants of the *Ec*-DHDPR structure show that the data most closely matches that expected for the β-10 (χ = 1.64) or β*-*8 dimers (χ = 1.50), and not the tetramer (χ = 4.86) or monomer (χ = 8.39) (Figure S6).

**Table 3:** Small angle x-ray scattering parameters.

	*Ec*-DHDPR		*Tm*-DHDPR		*At*-DHDPR2	
	Theoretical	Experimental	Theoretical	Experimental	Theoretical	Experimental
Envelope Volume	176,300*	182,129Λ	150,200*	152,975Λ	108,000∼	108,074Λ
Molecular weight (Da)	114,800 (tetramer)	113,830Λ	99,380 (tetramer)	95,609Λ	64,110 (dimer)	67,546Λ
Dmax (A)	–	107Λ	–	122Λ	–	114Λ
Rg (A)	31.3*	33.3#	31.4*	35.5#	–	33.3#

* calculated from 1arz for *Ec*-DHDPR and 1vm6 for *Tm*-DHDPR using CRYSOL;

Λ calculated from scattering data using AUTOPOROD;

# calculated from scattering data using GNOM;

∼ calculated from dimeric model generated using SWISS-MODEL and CORAL (see materials and methods).

**Figure 7:**
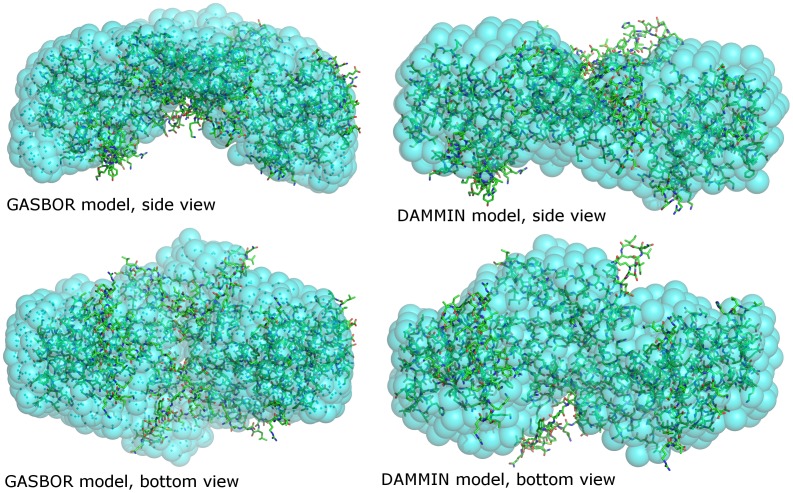
Results of *ab initio* modeling of *At*-DHDPR from SAXS data. Models were generated using GASBOR (left panels) and DAMMIN (right panels). The structural homology model generated by SWISS-MODEL and fitted to the scattering data using CORAL is superimposed for comparison.

In order to determine the arrangement of the *At*-DHDPR dimer, a structural homology model of *At*-DHDPR2 was generated using SWISS-MODEL [43], using the 1dih (*Ec*-DHDPR) structure as a template. The alignment had a low QMEAN score (−5.51) indicating a low quality of fit, suggesting that there are large structural differences between the plant and bacterial DHDPR structures. Much of the secondary structure was conserved in the model, however there were differences modelled in several of the loop regions, including the C-terminal loop and N-terminal loop regions that vary between different bacterial forms of the enzyme [23]–[24]. This model was used for rigid body refinement against the scattering data, which also modelled a 20 amino acid residue extension missing from the N-terminus of the input structure. In an independent approach *ab initio* bead modelling of the SAXS data reconstructed a shape that was elongated, and consistent with the refined rigid body model (Figure 7). However there was insufficient resolution to determine if the dimer was in the β-8 or β-10 arrangement, or had a novel dimeric interface.

**Table 4:** X-ray data collection and structure refinement statistics for *At*-DHDPS2 and lysine bound *At*-DHDPS2.

	*At-*DHDPS	*At-*DHDPS + Lysine
**Data collection**		
Space group	*P*4_1_2_1_2	*P*4_1_2_1_2
Wavelength (Å)	0.9537	0.9537
No. images	240	180
Oscillation range per image (°)	0.5	0.5
Detector	ADSC Quantum 315r	ADSC Quantum 315r
Detector distance (mm)	300	300
Exposure time (s)	1	3
Cell dimensions		
*a*, *b*, *c* (Å)	95.81, 95.81, 174.77	95.48, 95.48, 174.50
α, β, γ (°)	90, 90, 90	90, 90, 90
Resolution (Å)	37.00-2.00 (2.05 2.00)	36.64-2.20 (2.26-2.20)
*R*_sym_ †	0.096 (0.710)	0.121 (0.638)
*R*_pim_ ‡	0.033 (0.240)	0.049 (0.264)
*R*_rim_ §	0.102 (0.750)	0.131 (0.692)
*I/*δ*I*	17.4 (3.4)	13.3 (4.1)
Completeness (%)	100.0 (100.0)	99.9 (99.4)
Redundancy	9.6 (9.7)	7.1 (6.9)
Wilson *B-*factor (Å^2^)	25.69	25.61
Matthews Coefficient V_M_ (Å^3^ Da^−1^)	2.89	2.87
Solvent content (%)	57.5	57.1
**Refinement**		
Resolution (Å)	37.00-2.00 (2.05 2.00)	36.64-2.20 (2.26-2.20)
No. Reflections used in refinement	52873	39516
No. *R*_free_ reflections	2826	2090
*R*_work_	0.144	0.139
*R*_free_	0.181	0.176
Protein molecules in asymmetric unit	2	2
No. atoms	5177	5065
Protein	4737	4719
Ligand/ion	5	25
Water	435	321
Mean *B*-factor (Å^2^)	26.10	23.86
Protein	25.08	23.26
Ligand/ion	36.33	23.74
Water	37.09	32.75
R.m.s. deviations		
Bond lengths (Å)	0.018	0.018
Bond angles (°)	1.90	1.94

Values for the highest resolution shells are given in parentheses. The Matthew’s coefficient and estimate of the solvent content are based on 2 molecules of *At*-DHDPS2, 34 679.5 Da each, in the asymmetric unit.

† *R*_sym_ = ∑*_hkl_*∑*_i_* |*I_i_* (*hkl*)−〈*I(hkl)*〉|/∑*_hkl_*∑*_i_I_i_* (*hkl*).

‡ *R*_p.i.m._ = ∑*_hkl_* [1/(*N*−1)]^½^ ∑*_i_* |*I_i_* (*hkl*)−〈*I (hkl)*〉|/∑*_hkl_* ∑*_i_ I_i_* (*hkl*).

§ *R*_r.i.m._ = ∑*_hkl_* [*N*/(*N*−1)]^½^ ∑*_i_* |*I_i_* (*hkl*)−〈*I (hkl)*〉|/∑*_hkl_* ∑*_i_ I_i_* (*hkl*).

### Conclusions

Improvement of crop nutritional value and development of herbicides through targeting the lysine biosynthetic pathway requires an in-depth understanding of the structure and function of the DHDPS and DHDPR enzymes. The catalytic abilities of *At*-DHDPS2 and *At*-DHDPR2 are similar to other plant enzymes, but improved assay techniques through use of a coupled assay and more stringent substrate synthesis suggest that the kinetic parameters measured in this study more accurately reflect the actual values of the enzyme.

Overproduction of (*S*)-lysine in plants is currently carried out through the expression of a feedback insensitive DHDPS enzyme, such as that from *Corynebacterium glutamicum* [44]. An increased understanding of how plant DHDPS is inhibited by (*S*)-lysine opens the potential for the generation of a lysine insensitive plant variant for use in improved crop quality. *At*-DHDPS2 is tightly regulated through inhibition by very low concentrations of (*S*)-lysine, the end product of the pathway. However, unlike a previous study of the *Ns*-DHDPS enzyme that observed changes in the orientation of the subunits upon binding of (*S*)-lysine, X-ray crystallographic and small angle X-ray scattering studies showed that no large structural changes occurred upon binding of (*S*)-lysine to the allosteric site of *At*-DHDPS2. Indeed, the changes observed in the lysine bound crystal structure compared to the unliganded structure are confined to residues of the lysine binding pocket itself. The side chain of Trp116 undergoes a shift in rotamer upon lysine binding, closing down against the side chain of the bound lysine in a gate like action. Small rearrangements of the Glu147, His119, Ile120 and Arg146 side chains to accommodate the lysine molecule were also observed. However, these changes in side chain configurations were not propagated to other regions of the structure. We note that the resolution of the structures presented here (2.0–2.2 A) is considerably higher than that of the *Ns*-DHDPS structures previously published (∼2.8 A) [16]. Since the lysine binding sites of *At*-DHDPS2 are not situated close to crystal contacts, crystal packing arguments should not preclude observation of structural changes around this site. As such, we would expect to detect any significant conformational changes in the structure due to lysine binding.

Given the similarity of the unliganded and lysine bound crystal structures, it is possible that lysine binding significantly alters the dynamics of *At*-DHDPS2, resulting in allosteric inhibition. We have previously shown that protein dynamics have a considerable effect on the activity of *E. coli* DHDPS [19], [39]. Examination of the structural dynamics of *At*-DHDPS2 may shed further light on this idea in future studies.

Despite similar catalytic properties, the quaternary structure of *At*-DHDPR2 is strikingly different to the bacterial enzyme. Phylogenetic analysis of bacterial, cyanobacterial and plant DHDPR protein sequences shows several major clusters that generally align with lineages determined from 16 s ribosomal RNA genes, with plant DHDPR sequences forming an isolated cluster that do not share any clear lineages with bacterial or cyanobacterial genes (Figure S8) [5]. For each of the distinct bacterial and archaea clusters, a representative tetrameric structure is known (gamma proteobacteria, *Ec*-DHDPR; alpha proteobacteria, *Bartonella henselae*; actinobacteria, *Mt*-DHDPR; firmicutes *Sa*-DHDPR; archaeabacteria *Tm*-DHDPR), raising the likelihood that the ancestral DHDPR enzyme was a tetramer. In this study, we have used analytical ultracentrifugation, static light scattering and small angle X-ray scattering studies to unequivocally show that *At*-DHDPR2 exists as a dimer. The reasons for the differences in quaternary structure are unclear, given that the enzymes have similar catalytic abilities, and the subunit arrangement of plant DHDPR remains uncertain. DHDPS has a similarly divergent quaternary structure, with different tetrameric arrangements for plant and bacterial enzymes, with S. aureus existing as a dimeric enzyme. It will be interesting to extend the current studies of *A. thaliana* DHDPR to other plant DHDPR enzymes and also to the cyanobacterial DHDPR enzymes, as well as the chloroplast NAD(P)H dehydrogenase enzyme, which has high homology to DHDPR.

## Materials and Methods

### Materials

Unless otherwise stated, chemicals were obtained from Sigma Chemical Co. GE Biosciences, or Invitrogen. (*S*)-ASA was synthesised using the methods of Roberts [45], and was the kind gift of Andrew Muscroft-Taylor. Unless otherwise stated, enzymes were manipulated at 4°C or on ice.

### Cloning, Expression & Purification

Plasmids encoding AT3G60880 (*At*-DHDPS2) and AT3G59890 (*At*-DHDPR2) were obtained from the Arabidopsis Information Resource (TAIR), Carnegie Institution of Washington, Stanford CA. Primer pairs encoding the predicted 5′-3′ ends of the ORF were used to amplify the gene. Primers were designed to exclude the chloroplast transit peptide, as identified using chloroP [46]. The PCR product was ligated into the pET151/D-Topo vector (Invitrogen), with reactions carried out according to the manufacturer’s protocols. Protein expression was performed in BL21(DE3) Star cells, using ZYM-5052 media [47]. Cultures were grown at 37°C for five hours, followed by incubation at 26°C overnight. Cells were harvested by centrifugation, resuspended in buffer containing 50 mM NaH_2_PO_4_, pH 8.0, 30 mM imidazole and 300 mM NaCl and lysed by sonication. Cell debris was pelleted by centrifugation, and the cell pellet applied to a His-Trap Crude column (GE Biosciences). The column was washed with three volumes of resuspension buffer, before bound protein was eluted using 50 mM NaH_2_PO_4_, pH 8.0, 300 mM imidazole and 300 mM NaCl. Cleavage of the His-tag was carried out by incubation of the enzyme with the TEV protease for two hours at 20°C, followed by removal of the cleaved tag using a His-trap column. Fractions containing protein were desalted into 20 mM Tris.HCl, pH 8.0 for storage.

### Size-Exclusion Chromatography

Gel filtration was carried out at 28°C using a Malvern P3000 column. 100 µL of enzyme (1.0 mg.mL^−1^) was loaded onto the column and eluted with 20 mM Tris-HCl, 150 mM NaCl, pH 8.0 at 0.5 mL.min^−1^. A Viscotek TDA unit was used to measure the refractive index and low angle and right angle light scattering. BSA (2 mg.mL^−1^) was used as a standard to calibrate the instrument.

### Analytical Ultracentrifugation

Sedimentation velocity experiments were performed in a Beckman Coulter Model XL-I analytical ultracentrifuge equipped with UV/Vis scanning optics. Reference (380 µL; 20 mM Tris-HCl, 150 mM NaCl, pH 8.0) and sample (360 µL) solutions were loaded into 12 mm double-sector cells with quartz windows and the cells were then mounted in an An-60 Ti 4-hole rotor. *At-*DHDPS2 was prepared at a concentration of 0.75 mg.mL^−1^ while *At-*DHDPR2 was prepared at initial protein concentrations of 0.1–1.6 mg.mL^−1^. Proteins were centrifuged at 40,000 rpm (*At-*DHDPR2) or 50,000 rpm (*At-*DHDPS2) at 20°C, and radial absorbance data were collected at appropriate wavelengths in continuous mode every 8 minutes without averaging. Data were fitted to a continuous size-distribution [*c(s)*] model using the program SEDFIT [48]. The partial specific volume (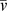) of the proteins (*At-*DHDPS2 0.738 mL g^−1^; *At-*DHDPR2 0.745 mL g^−1^), buffer density (1.005 g.mL^−1^) and buffer viscosity (1.021 cp) were computed using the program SEDNTERP [49].

### SAXS Measurements

Measurements were performed at the Australian Synchrotron SAXS/WAXS beamline equipped with a Pilatus detector (1 M, 170 mm×170 mm, effective pixel size, 172×172 µm). The wavelength of the X-rays was 1.0332 Å. The sample–detector distance was 1600 mm, which provided a *q* range of 0.0126–0.400 Å^−1^ [where *q* is the magnitude of the scattering vector, which is related to the scattering angle (*2θ*) and the wavelength (λ) as follows: *q* = (4π/λ)sinθ]. Protein samples (initial concentration of 2–5 mg mL^−1^) were eluted from an in-line gel filtration column (Superdex 200 5/150), pre-equilibrated with 20 mM Tris.HCl, 150 mM NaCl, pH 8.0, to remove any aggregated protein immediately prior to data collection. Data were collected using a 1.5 mm glass capillary at 27°C under continuous flow in 2 sec intervals. 2D intensity plots from the peak of the SEC run were radially averaged, normalized to sample transmission and background subtracted.

*SAXS data analysis*: The data sets for structural analyses were recorded with 458 data points over the range 0.0113≤ *s* ≤0.4 Å^−1^. 1D profiles were background subtracted and Guinier analysis performed using PRIMUS [50]. Particle volume and molecular weight were calculated using AUTOPOROD [51]. Indirect Fourier transform was performed using GNOM [52] to yield the real-space function *P(r)*, which gives both the relative probabilities of distances between scattering centers and the maximum dimension of the scattering particle *D*_max_. Theoretical scattering curves were calculated from atomic coordinates and compared with experimental scattering curves using CRYSOL [53]. For *At*-DHDPR a homology model for *At*-DHDPR2 was generated using SWISS-MODEL [43], which aligned the sequence to the 1dih structure. Rigid body refinement of this model was carried out using CORAL [54], additionally refining the 20 amino acid residues that were missing from the N-terminus of the SWISS-MODEL structure. *Ab initio* modeling was carried out using DAMMIN [55] to generate 10 bead models with imposed P2 symmetry. These models were averaged using DAMAVER, and superimposed with the bacterial structures using SUPCOMB [56]. GASBOR [57] was also used for *ab initio* reconstruction of of a dummy-residue model, using an extended data range relative to the short range used for bead-modeling.

### Crystallization, X-ray Diffraction Data Collection, and Structure Solution

*At*-DHDPS was crystallized essentially as described previously [37] using the sitting-drop vapor diffusion method. The crystals used for diffraction analysis and structure solution were obtained at 20°C from 300 nL drops formed from 150 nL *At*-DHDPS2 solution (14.5 mg.mL^−1^ in 20 mM Tris-HCl pH 8.0) and 150 nL reservoir solution [2.4 M sodium malonate pH 7.0, 0.02% (w/v) sodium azide]. Ligand soaks were performed by adding 300 nL of 10 mM lysine, 2.4 M sodium malonate pH 7.0, 0.02% (w/v) sodium azide directly to the crystallization drop followed by incubation for 2 hours. The malonate concentration present in the crystallization drop was found to afford adequate cryo-protection and, thus, crystals were flash-cooled in liquid nitrogen directly from the crystallisation drop. X-ray diffraction data collection was carried out at 110 K at using the MX2 beam line of the Australian Synchrotron.

Diffraction data sets were processed and scaled using the programs XDS [58] and SCALA [59]. Initial phase estimates were solved by molecular replacement using PHASER [60] with the lysine bound structure of *N. sylvestris* DHDPS [16] as the search model as described previously [37]. Structural refinement was performed using REFMAC5 [61] with iterative model building using COOT [62]. Water, sodium ions, and the bound lysine atoms were added at later stages using COOT. Data processing and structure refinement statistics are presented in Table 4.

### Enzyme Kinetics

DHDPS and DHDPR enzyme activity was measured using a coupled assay as previously described. Stock solutions of (*S*)-ASA, pyruvate, (*S*)-lysine, NADPH and NADH were prepared fresh for each experiment. Assay temperature was regulated by the use of a circulating water bath, and assays were performed at 30°C. Initial rate data were typically reproducible within 10%, and were analysed using non-linear regression software (OriginLab, Northampton, MA, USA). Assays for DHDPS activity contained 0.5 µg.mL^−1^ DHDPS, and an excess of DHDPR (20–100 µg.mL^−1^). Assays for DHDPR activity involved pre-incubating the cuvettes with an excess of DHDPS (20–100 µg.mL^−1^) for 60 s before assays were initiated by the addition of DHDPR (typically 0.6 µg.mL^−1^).

## Supporting Information

Figure S1 **Lysine biosynthesis pathways.** DapD, tetrahydrodipicolinate acylase; DapC,acyl-amino-ketopimelate aminotransferase; DapE, acyl-ketopimelate deacylase; DapF, diaminopimelate epimerase; LysA, diaminopimelate decarboxylase; DapDH, mesodiaminopimelatedehydrogenase; DapL, l,l-diaminopimelate aminotransferase.
(PDF)

Figure S2 **Kinetics of** ***At*** **-DHDPS2.** Assays were carried out at varying concentrations of (S)-lysine (top panel), or varying concentrations of ASA and pyruvate (bottom panel).
(PDF)

Figure S3 **Kinetics of** ***At*** **-DHDPR2.** Panel A) HTPA was fixed at 0.15 mM, and NAD(PH concentrations were varied. Panel B) NAD(P)H concentrations were fixed at 0.16 mM and HTPA concentrations were varied.
(PDF)

Figure S4 **Structure of bacterial DHDPR.** The structure of *Ec*-DHDPR (pdb: 1arz), showing the interface between β-8 and loop 1 (left panel) and the alternate interface involving β-10 and helix-4 (right panel, only the C-terminal domain shown).
(PDF)

Figure S5 **Alignment of DHDPR structures**. Helical regions are shown in blue and β-sheet regions are shown in red.
(PDF)

Figure S6 **X-Ray scattering of** ***At*** **-DHDPR2**. Data was collected and compared to the scattering calculated using CRYSOL for the monomer, β-10 dimer, β-8 dimer, and tetramer of *Ec*-DHDPR.
(PDF)

Figure S7 **Residuals resulting from the** ***c(s)*** **distribution best fits shown in** **Figure 2** **plotted as a function of radius from the axis of rotation.** A) Residuals for the best fit of the sedimentation velocity data for *At-*DHDPS2 at a concentration of 0.75 mg.mL^−1^. B) Residuals for the best fit of the sedimentation velocity data for *At-*DHDPR2 at concentrations of 0.1 mg.mL^−1^ (black), 0.2 mg.mL^−1^ (red), 0.4 mg.mL^−1^ (green), 0.8 mg.mL^−1^ (pink), and 1.6 mg.mL^−1^ (blue).
(PDF)

Figure S8 **Representative phylogenetic tree of DapB orthologues based on Hudson, 2005** [**5**] **.**
(PDF)
