# Supplementary material for: Characterisation of the First Enzymes Committed to Lysine Biosynthesis in Arabidopsis thaliana
Source: PLoS One. 2012 Jul 5;7(7):e40318. doi: 10.1371/journal.pone.0040318 (PMC3390394; doi:10.1371/journal.pone.0040318)
Supplement: Figure S1 — Lysine biosynthesis pathways. DapD, tetrahydrodipicolinate acylase; DapC,acyl-amino-ketopimelate aminotransferase; DapE, acyl-ketopimelate deacylase; DapF, diaminopimelate epimerase; LysA, diaminopimelate decarboxylase; DapDH, mesodiaminopimelatedehydrogenase; DapL, l,l-diaminopimelate aminotransferase. (PDF) [file pone.0040318.s001.pdf]

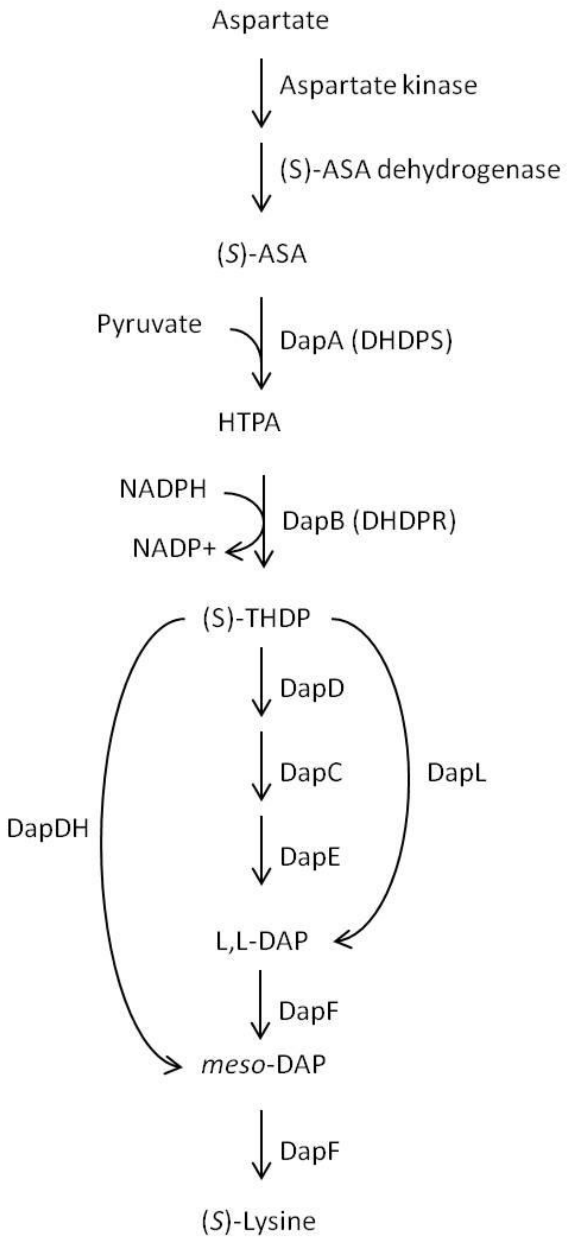

Figure S1: Lysine biosynthesis pathways. DapD, tetrahydrodipicolinate acylase; DapC,acyl-amino-ketopimelate aminotransferase; DapE, acyl-ketopimelate deacylase; DapF, diaminopimelate epimerase; LysA, diaminopimelate decarboxylase; DapDH, mesodiaminopimelatedehydrogenase; DapL, l,l-diaminopimelate aminotransferase.
