# Supplementary material for: Characterisation of the First Enzymes Committed to Lysine Biosynthesis in Arabidopsis thaliana
Source: PLoS One. 2012 Jul 5;7(7):e40318. doi: 10.1371/journal.pone.0040318 (PMC3390394; doi:10.1371/journal.pone.0040318)
Supplement: Figure S2 — Kinetics of At -DHDPS2. Assays were carried out at varying concentrations of (S)-lysine (top panel), or varying concentrations of ASA and pyruvate (bottom panel). (PDF) [file pone.0040318.s002.pdf]

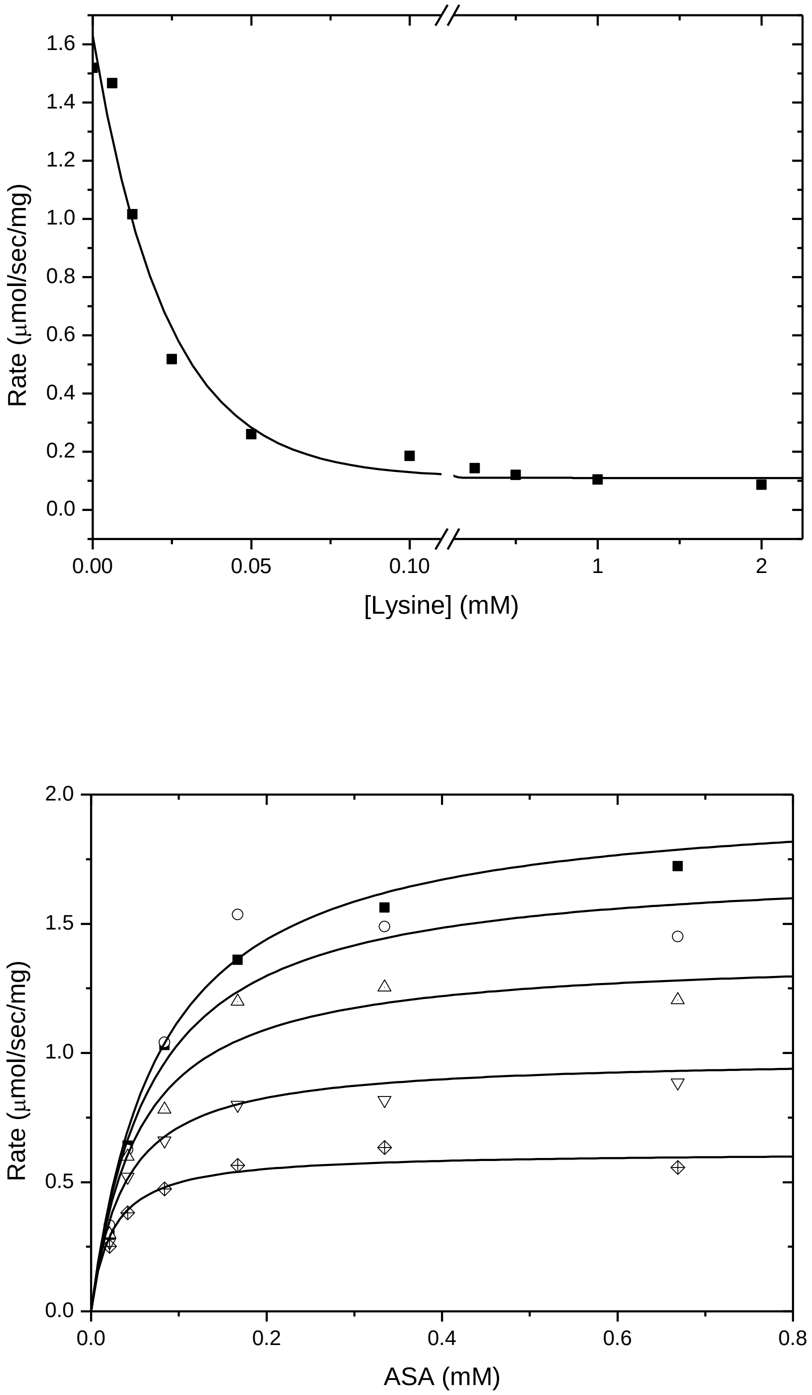

Fig S2: Kinetics of *At*-DHDPS2. Assays were carried out at varying concentrations of (S)-lysine (top panel), or varying concentrations of ASA and pyruvate (bottom panel).
