# Supplementary material for: Characterisation of the First Enzymes Committed to Lysine Biosynthesis in Arabidopsis thaliana
Source: PLoS One. 2012 Jul 5;7(7):e40318. doi: 10.1371/journal.pone.0040318 (PMC3390394; doi:10.1371/journal.pone.0040318)
Supplement: Figure S3 — Kinetics of At -DHDPR2. Panel A) HTPA was fixed at 0.15 mM, and NAD(PH concentrations were varied. Panel B) NAD(P)H concentrations were fixed at 0.16 mM and HTPA concentrations were varied. (PDF) [file pone.0040318.s003.pdf]

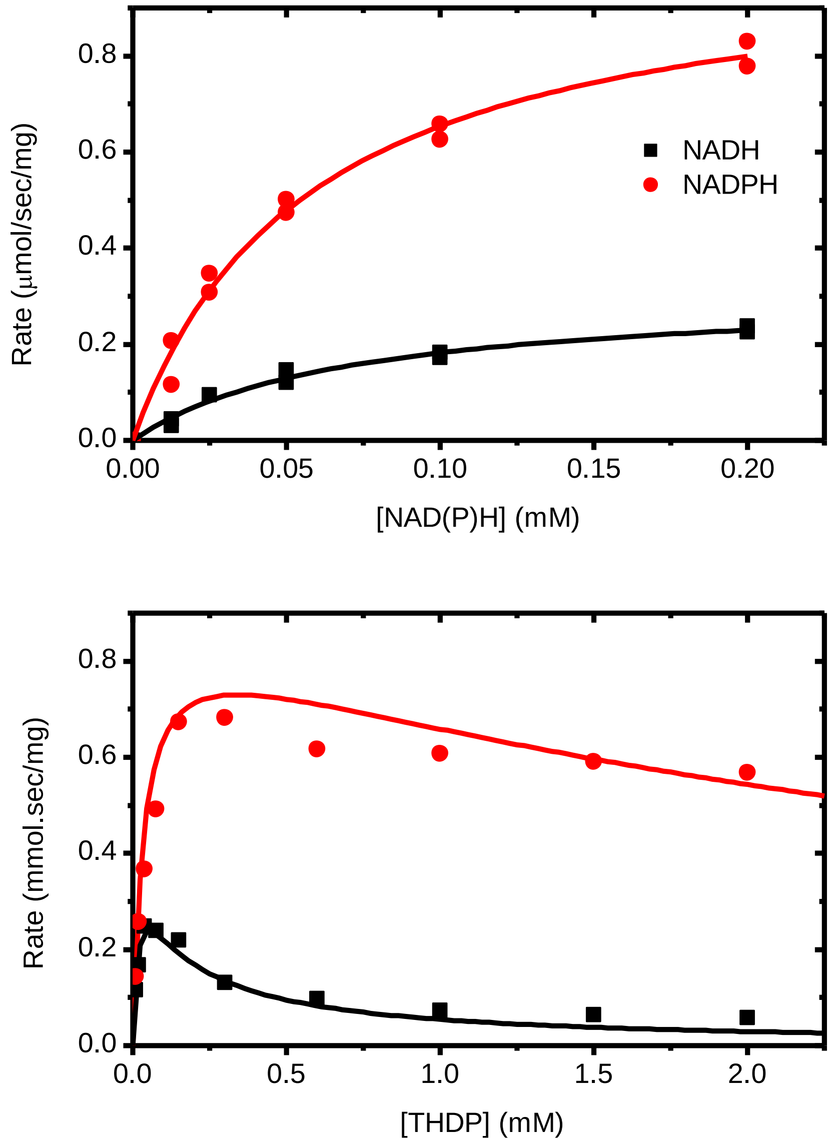


**Figure S3: Kinetics of *At*-DHDPR2.** Panel A) HTPA was fixed at 0.15 mM, and NAD(PH concentrations were varied. Panel B) NAD(P)H concentrations were fixed at 0.16 mM and HTPA concentrations were varied.
