# Supplementary material for: Characterisation of the First Enzymes Committed to Lysine Biosynthesis in Arabidopsis thaliana
Source: PLoS One. 2012 Jul 5;7(7):e40318. doi: 10.1371/journal.pone.0040318 (PMC3390394; doi:10.1371/journal.pone.0040318)
Supplement: Figure S4 — Structure of bacterial DHDPR. The structure of Ec-DHDPR (pdb: 1arz), showing the interface between β-8 and loop 1 (left panel) and the alternate interface involving β-10 and helix-4 (right panel, only the C-terminal domain shown). (PDF) [file pone.0040318.s004.pdf]

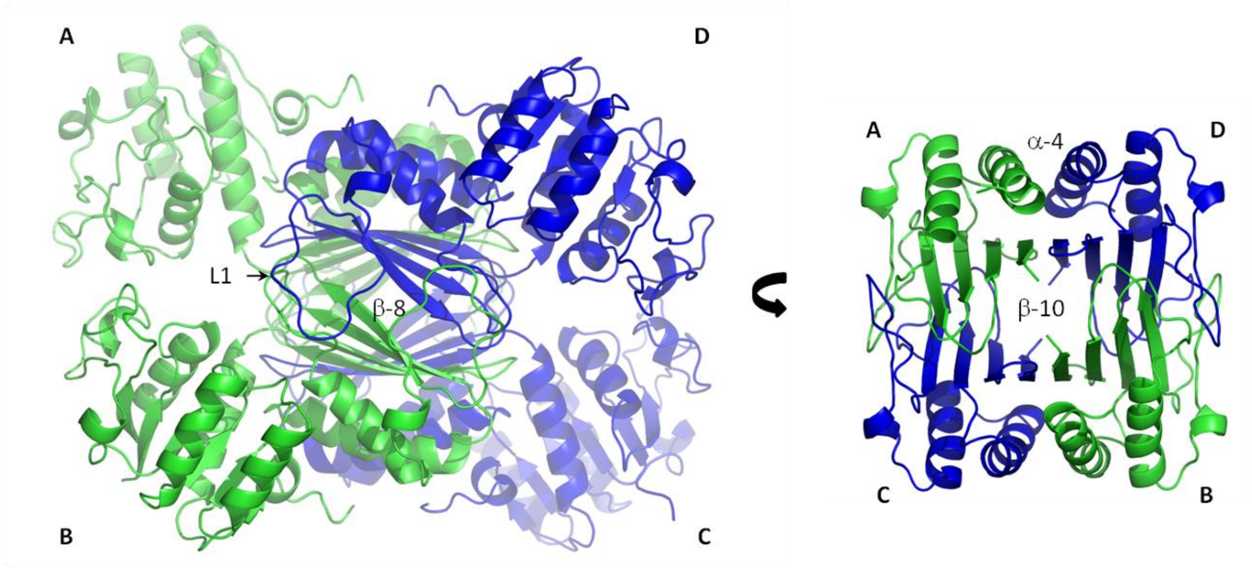

Figure S4: Structure of bacterial DHDPR. The structure of *Ec*-DHDPR (pdb: 1arz), showing the interface between β-8 and loop 1 (left panel) and the alternate interface involving β-10 and helix-4 (right panel, only the C-terminal domain shown).
