# Supplementary material for: Characterisation of the First Enzymes Committed to Lysine Biosynthesis in Arabidopsis thaliana
Source: PLoS One. 2012 Jul 5;7(7):e40318. doi: 10.1371/journal.pone.0040318 (PMC3390394; doi:10.1371/journal.pone.0040318)
Supplement: Figure S5 — Alignment of DHDPR structures. Helical regions are shown in blue and β-sheet regions are shown in red. (PDF) [file pone.0040318.s005.pdf]

```
EC_DHDPR        MH----------------DANIRVAIAGAGGRMGRQLIQAALALEGVQLG
MT_DHDPR        ---------------------MRVGVLGAKGKVGATMVRAVAA-A-DDLT
TM_DHDPR        MGSDK---IH------HHHHHMKYGIVGYSGRMGQEIQKVFS-EKGHELV
SA_DHDPR        M------------------ASMKILLIGY-GAMNQRVARLAE-EKGHEIV
AT_DHDPR1       AMAD---SGEEAVKSVLPGNGISIMVNGCSGKMGKAVIKAAD-SAGVNIV
AT_DHDPR2       LSMGKSETFEEAGNSVAPGNGISIMVNGCSGKMGKAVIKAAD-SAGVNIV
                                     :   : *  * :.  : :        ::

EC_DHDPR        AALER----EGSSLLGSDAGELAGAGKTGVTVQSSLDAV--KDDFDVFID
MT_DHDPR        LSAE-------------------------LDAGDPLSLLT-DGNTEVVID
TM_DHDPR        LKVD---------------------------V-NGVEE---LDSPDVVID
SA_DHDPR        GVIEN-----------------TPKAT--TPY-QQYQHIADVKGADVAID
AT_DHDPR1       PISFGSAGEDGQ-RVEVCGKEITVHGP--TEREKVLSSVFEKHPELIVVD
AT_DHDPR2       PTSFGSVEEAGQ-TVEVCGKEILVHGP--TEREKVLSSVFEKYPELIVVD
                                                 .  .         : :*

EC_DHDPR        FTRPEGTLNHLAFCRQHGKGMVIGTTGFDEAGKQAIRDA---AADIAIVF
MT_DHDPR        FTHPDVVMGNLEFLIDNGIHAVVGTTGFTAERFQQVESWLVAKPNTSVLI
TM_DHDPR        FSSPEALPKTVDLCKKYRAGLVLGTTALKEEHLQMLREL---SKEVPVVQ
SA_DHDPR        FSNPNLLFPLL--DEDFHLPLVVATTGEKEKLLNKLDEL---SQNMPVFF
AT_DHDPR1       YTIPSAVNDNAELYSKVGVPFVMGTTGGDRNKLYETVEE---AKIYA-VI
AT_DHDPR2       YTIPSAVNDNAELYGKVGVPFVMGTTGGDRTRLYKTVEE---SKIYA-VI
                :: *.          .     *:.**.          .        . .

EC_DHDPR        AANFSVGVNVM---LKLLEKAA-KVMGDYTDIEIIEAHHRHKVDAPSGTA
MT_DHDPR        APNFAIGAVLS---MHFAKQAA-RFFD---SAEVIELHHPHKADAPSGTA
TM_DHDPR        AYNFSIGINVL---KRFLSELV-KVLEDW-DVEIVETHHRFKKDAPSGTA
SA_DHDPR        SANMSYGVHAL---TKILAAAV-PLLDDF-DIELTEAHHNKKVDAPSGTL
AT_DHDPR1       SPQMGKQVVAFLAAMEIMAEQFPGAFSGY-SLDVMESHQASKLDA-SGTA
AT_DHDPR2       SPQMGKQVVAFLAAMEIMSEQFPGAFAGY-SLEVMESHQASKLDA-SGTA
                : ::.          .:        :    . :: * *:  * **  ***

EC_DHDPR        LAMGEAIAHAL---DKDLKDCAVYSREGHTGERVP-GTIGFATVRAGDIV
MT_DHDPR        ARTAKLIAEARKGLPPNP-DATSTSLPGARGADV--DGIPVHAVRLAGLV
TM_DHDPR        ILLESALGKS---------------------------VPIHSLRVGGVP
SA_DHDPR        EKLYDVIVSLKENVTPVY-DRHELNE-----KRQP-QDIGIHSIRGGTIV
AT_DHDPR1       KAVISCFQ----ELGVSY-DMDQIQL-----IRDPKQQVEMVGVPEEHIS
AT_DHDPR2       KAVISCFQ----KLGVSY-DMDQIQL-----IRDPKQQIEVVGVPEEHVS
                    . :                               : .  :    :

EC_DHDPR        GE--HTAMFADIG--ERLEITHKASSRMTFANGAVRSALWLSGK-----E
MT_DHDPR        AH--QEVLFGIEG--ETLTIRHDSLDRTSFVPGVLLAVRRIAER-----P
TM_DHDPR        GD--HVVVFGNIG--ETIEIKHRAISRTVFAIGALKAAEFLVGK-----D
SA_DHDPR        GE--HEVLFAGTD--ETIQITHRAQSKDIFANGAIQAAERLVNK-----P
AT_DHDPR1       GHAFHLYHLTSPDETVSFEFQHNVCGRSIYAEGTVDAVLFLAKKIRLKAD
AT_DHDPR2       GHAFHLYHLTSPDKTVSFEFQHNVCGRSIYAEGTVDAVLFLAKKIRSKAE
                ..  :   :   .    : : *   .:  :. *.: :.  :  :

EC_DHDPR        SGLFDMRDVL-DLNNL
MT_DHDPR        -GLTVGLEPL--LDLH
TM_DHDPR        PGMYSFEEVI--FGGE
SA_DHDPR        NGFYT-------FDNL
AT_DHDPR1       QRIYNMIDVLREGNMR
AT_DHDPR2       KRIYNMIDVLREGNMR
                  :          .
```

Figure S5: Alignment of DHDPR sequences. Helical regions are shown in blue and β-sheet regions are shown in red.
