# Supplementary material for: Characterisation of the First Enzymes Committed to Lysine Biosynthesis in Arabidopsis thaliana
Source: PLoS One. 2012 Jul 5;7(7):e40318. doi: 10.1371/journal.pone.0040318 (PMC3390394; doi:10.1371/journal.pone.0040318)
Supplement: Figure S6 — X-Ray scattering of At -DHDPR2. Data was collected and compared to the scattering calculated using CRYSOL for the monomer, β-10 dimer, β-8 dimer, and tetramer of Ec-DHDPR. (PDF) [file pone.0040318.s006.pdf]

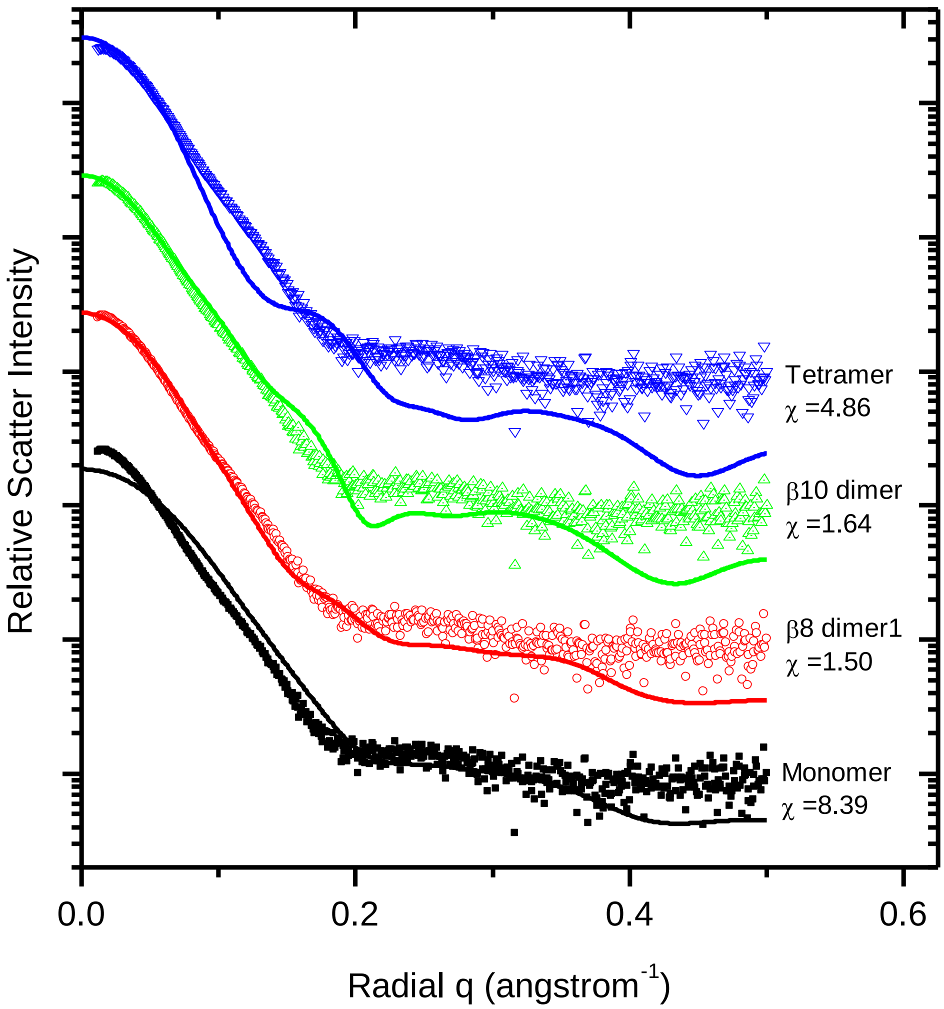

Figure S6: X-Ray scattering of *At*-DHDPR2. Data was collected and compared to the scattering calculated using CRYSOL for the monomer, β-10 dimer, β-8 dimer, and tetramer of *Ec*-DHDPR.
