# Supplementary material for: Characterisation of the First Enzymes Committed to Lysine Biosynthesis in Arabidopsis thaliana
Source: PLoS One. 2012 Jul 5;7(7):e40318. doi: 10.1371/journal.pone.0040318 (PMC3390394; doi:10.1371/journal.pone.0040318)
Supplement: Figure S7 — Residuals resulting from the c(s) distribution best fits shown in Figure 2 plotted as a function of radius from the axis of rotation. A) Residuals for the best fit of the sedimentation velocity data for At-DHDPS2 at a concentration of 0.75 mg.mL−1. B) Residuals for the best fit of the sedimentation velocity data for At-DHDPR2 at concentrations of 0.1 mg.mL−1 (black), 0.2 mg.mL−1 (red), 0.4 mg.mL−1 (green), 0.8 mg.mL−1 (pink), and 1.6 mg.mL−1 (blue). (PDF) [file pone.0040318.s007.pdf]

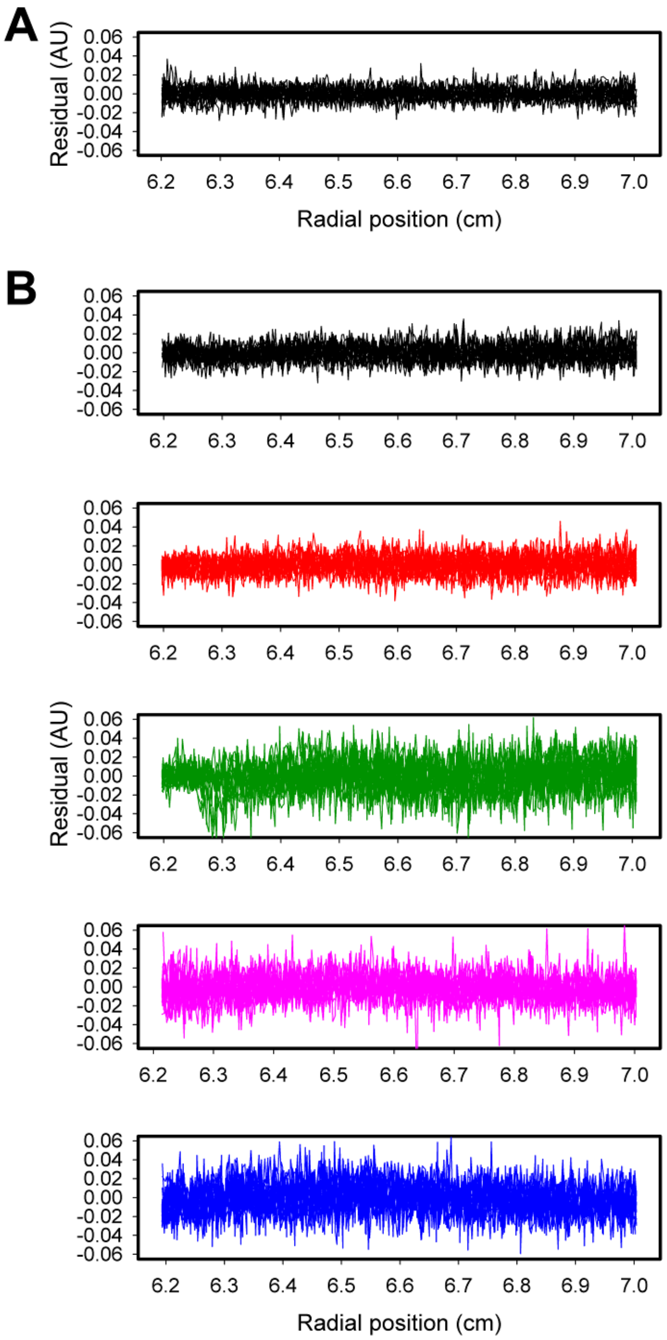

Figure S7. Residuals resulting from the *c(s)* distribution best fits shown in Figure 2 plotted as a function of radius from the axis of rotation. A) Residuals for the best fit of the sedimentation velocity data for *At*-DHDPS2 at a concentration of 0.75 mg.mL^{-1}. B) Residuals for the best fit of the sedimentation velocity data for *At*-DHDPR2 at concentrations of 0.1 mg.mL^{-1} (black), 0.2 mg.mL^{-1} (red), 0.4 mg.mL^{-1} (green), 0.8 mg.mL^{-1} (pink), and 1.6 mg.mL^{-1} (blue).
