# Supplementary figures and images for: Characterisation of the First Enzymes Committed to Lysine Biosynthesis in Arabidopsis thaliana
Source: PLoS One. 2012 Jul 5;7(7):e40318. doi: 10.1371/journal.pone.0040318 (PMC3390394; doi:10.1371/journal.pone.0040318)

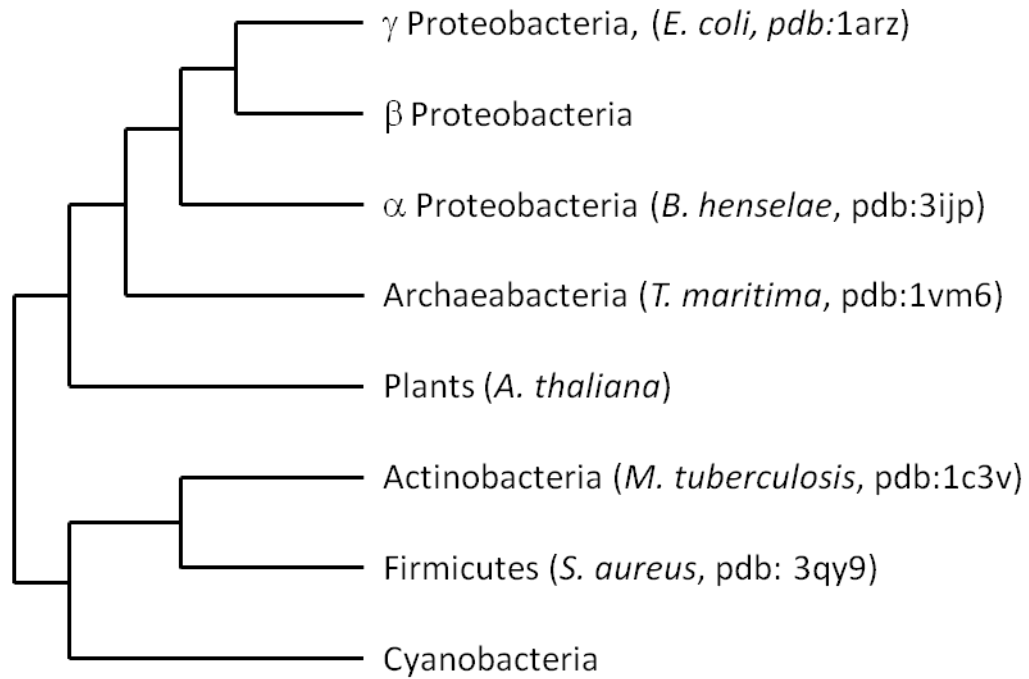

**Figure S8:** Representative phylogenetic tree of DapB orthologues based on Hudson, 2005 [5].

Supplement: Figure S8 — Representative phylogenetic tree of DapB orthologues based on Hudson, 2005 [5] . (PDF) [file pone.0040318.s008.pdf]
